# General inverse-cube thickness scaling of projectile penetration energy in ultrathin films

**DOI:** 10.1073/pnas.2609202123

**Published:** 2026-06-10

**Authors:** Alessio Zaccone, Timothy W. Sirk

**Affiliations:** ^a^ Department of Physics “A. Pontremoli”, University of Milan, Milan 20133, Italy; ^b^ Polymers Branch, DEVCOM Army Research Laboratory, Aberdeen Proving Ground, Aberdeen, MD 21005

**Keywords:** projectile impact, thin films, nanoscale confinement, nonaffine elasticity, 2D materials

## Abstract

Ultrathin films of widely different materials exhibit a dramatic enhancement of projectile penetration resistance under high–velocity impact. Despite extensive simulations and experiments, a unifying physical explanation has remained elusive. Here we show that the specific penetration energy follows a general inverse-cube scaling law, $E_{p}^{*}\left(h\right)=E_{p,\infty }^{*}+Bh^{-3}$, across chemically and structurally distinct systems. The inverse–cube scaling is traced to a finite–size correction to the effective shear modulus caused by suppression of long–wavelength nonaffine modes. The scaling describes impact data for multilayer graphene, graphene oxide, and polymer thin films, revealing a common elastic contribution to nanoscale impact resistance.

Under high–velocity impact in the elastic–inertial regime, projectile resistance is governed by stress-wave propagation and momentum transfer within the target. The relevant material parameter controlling the attainable transient shear stresses is the effective high-rate shear modulus. For nanometric films, this modulus is strongly affected by confinement, which suppresses long–wavelength deformation modes. In real (disordered and partially ordered) solids, these modes are predominantly nonaffine and contribute negatively to the shear modulus. These modes are internal atomic or molecular motions, not global bending: Classical bending gives $D\propto Eh^{3}$ and would predict the opposite trend (1).

The mechanical response of real-world solids differs fundamentally from that of perfect crystalline materials, due to the presence of nonaffine particle displacements. At the microscopic level, the equation of motion for the displacement **x** of particle *i* under an applied strain can be derived from a system–bath Caldeira–Leggett Hamiltonian and reads (2, 3)

[1] $$\begin{array}{c} m{\ddot{x}}_{i}+\nu {\dot{x}}_{i}+\sum_{j}H_{\mathrm{ij}}x_{j}=\Xi_{i,k\ell }\;\eta_{k\ell }, \end{array}$$

where *m* is the particle mass, *ν* an interatomic friction coefficient, $H_{\mathrm{ij}}$ the Hessian (dynamical matrix) describing harmonic nearest-neighbor interactions, $\eta_{k\ell }$ the applied strain tensor (kℓ=xy for shear), and $\Xi_{i,k\ell }$ the affine force vector field generated by the lack of local inversion symmetry (due to defects, thermal motion, or disorder). In real systems, the force on each atom does not vanish in an affine deformation, requiring additional nonaffine relaxations that reduce the shear modulus (4).

Solving Eq. **1** in Fourier space and projecting onto the eigenvectors of the Hessian matrix, further manipulations yield the complex shear modulus (2, 5)

[2] $$\begin{array}{c} G^{*}\left(\omega \right)=G_{\infty }-A\sum_{k,\lambda }\frac{\omega_{k,\lambda }^{2}}{\omega_{k,\lambda }^{2}-\omega^{2}+i\omega \nu }, \end{array}$$

where $G_{\infty }$ is the affine (Born) shear modulus that survives in the infinite frequency limit, $\omega_{k,\lambda }$ are the eigenfrequencies of the vibrational modes indexed by wavevector **k** and polarization *λ*, and the second term represents the softening contribution from nonaffine relaxations. Following nonaffine lattice dynamics (2, 3, 5), the complex shear modulus can be written as a sum over vibrational eigenmodes

[3] $$\begin{array}{c} G^{*}\left(\omega \right)=G_{\infty }-\frac{1}{V}\sum_{p}\frac{\Gamma_{p}\left(\omega_{p}\right)}{\omega_{p}^{2}-\omega^{2}+i\omega \nu }, \end{array}$$

where $G_{\infty }$ is the affine (Born) modulus, $\omega_{p}$ are eigenfrequencies, *ν* is a microscopic damping coefficient, and $\Gamma_{p}\geq 0$ are mode coupling weights (set by projections of the nonaffine force field onto mode *p*). In the quasistatic limit ω→0 one obtains $G^{\prime }\left(\omega \;\to \;0\right)=G_{\infty }-G_{\mathrm{NA}}$ where the extent of the residual nonaffine contribution $G_{\mathrm{NA}}$ depends on the type of chemical bonding and/or molecular forces, and on the atomic-scale geometric environment (it tends to vanish only for perfectly centrosymmetric crystals at low temperature).

We consider a thin film of thickness $L\equiv L_{z}$ with lateral dimensions $L_{x},L_{y}\gg L$ (i.e. $L\equiv L_{z}\ll L_{x},L_{y}$). In a finite system the allowed wavevectors are discrete, and the smallest accessible magnitude is set by the system dimensions. In particular,

[4] $$\begin{array}{c} k_{\min}\equiv \left|k_{\min}\right|=2\pi \sqrt{{\left(\frac{1}{L_{x}}\right)}^{2}+{\left(\frac{1}{L_{y}}\right)}^{2}+{\left(\frac{1}{L}\right)}^{2}}\;∼\;\frac{1}{L}, \end{array}$$

so that in the thin-film limit the infrared cut-off is controlled by the thickness. For a rigorous justification of this relation, including the exact prefactors, see ref. 6.

Following refs. 1 and 6, we replace the discrete sum over modes by a momentum integral. At low frequencies the nonaffine softening contribution can be represented in the form

[5] $$\begin{array}{c} G^{\prime }=G_{\infty }-\alpha \int_{k_{\min}}^{k_{D}}k^{2}\;dk, \end{array}$$

where $G_{\infty }$ is the affine (Born) modulus, $k_{D}$ is a Debye-like ultraviolet cutoff, and α>0 collects material-dependent prefactors (including mode-coupling weights). Performing the integral gives

[6] $$\begin{array}{c} G^{\prime }=G_{\infty }-\frac{\alpha }{3}\left(k_{D}^{3}-k_{\min}^{3}\right)=\underset{\equiv \;G_{\mathrm{bulk}}^{\prime }}{\underbrace{\left(G_{\infty }-\frac{\alpha }{3}k_{D}^{3}\right)}}+\frac{\alpha }{3}k_{\min}^{3}. \end{array}$$

Using Eq. **4**, $k_{\min}∼1/L$, one obtains the inverse-cube finite-size correction

[7] $$\begin{array}{c} G^{\prime }\left(L\right)=G_{\mathrm{bulk}}^{\prime }+\frac{\beta }{3}\;L^{-3},\;\beta \equiv \alpha , \end{array}$$

which is the explicit confinement-induced $L^{-3}$ scaling reported in Eq. (**6**) of Phillips et al. (6). Identifying *L* with the film thickness *h* yields

[8] $$\begin{array}{c} G^{\prime }\left(h\right)=G_{\mathrm{bulk}}^{\prime }+\beta^{\prime }\;h^{-3}, \end{array}$$

with $\beta^{\prime }=\beta /3$ after absorbing numerical factors into the material-dependent prefactor. This continuum derivation is asymptotic; for few-layer graphene, $h^{-3}$ should be read as an effective scaling, with possible deviations at very small *N* (Table 1).

**Table 1.** Fitted values of the finite-size coefficient *B* in $E_{p}^{*}\left(x\right)$ = $E_{p,\infty }^{*}$ + $Bx^{-3}$ for the three systems shown in Fig. 1

System	Variable *x*	*B*	Units
Multilayer graphene	*N*	2.6 × 10^1^	MJ/kg
Graphene oxide films	*h*	4.9 × 10^2^	MJ nm^3^/kg
Polymer thin films	*h*	7.8 × 10^5^	MJ nm^3^/kg

In the elastic–inertial regime of high-velocity penetration, projectile resistance is governed by stress-wave propagation and momentum transfer within the target. This framework does not attempt to replace ballistic-limit models that include strength and failure criteria (e.g., Phoenix-type models), but instead identifies the physical origin of the observed thickness scaling within the elastic stiffness entering such models. Thus $E_{p}^{*}\left(h\right)\propto G^{\prime }\left(h\right)$ is a scaling argument for the elastic contribution, with fracture, plasticity, and dissipation adding further terms. The characteristic stress scale generated around the projectile boundary scales with the transverse wave impedance. The transverse sound speed satisfies $c_{T}∼\sqrt{G^{\prime }/\rho }$, and the corresponding impedance is $Z∼\rho c_{T}∼\sqrt{\rho G^{\prime }}$. For comparable projectile geometry and impact velocity, the attainable shear stress and the rate of momentum transfer therefore inherit the thickness dependence of $G^{\prime }\left(h\right)$. Consequently, within this elastic–inertial scaling description, the thickness dependence of the specific penetration energy follows that of the shear modulus, so that $E_{p}^{*}\left(h\right)\propto G^{\prime }\left(h\right)$ (7–9). Combining with Eq. **8** gives

[9] $$\begin{array}{c} E_{p}^{*}\left(h\right)=E_{p,\infty }^{*}+B\;h^{-3}, \end{array}$$

where $E_{p,\infty }^{*}\propto G_{\mathrm{bulk}}^{\prime }$ and $B\propto \beta^{\prime }/\rho$. In practice, microscopic length scales introduce a small cutoff $h_{0}$, leading to a regularized form $E_{p}^{*}\left(h\right)=E_{p,\infty }^{*}+B{\left(h+h_{0}\right)}^{-3}$ that avoids divergence as h→0 (10). The cutoff $h_{0}$ represents a microscopic length below which continuum scaling breaks down; it is negligible for the thickness range considered.

We emphasize that $E_{p}^{*}$ is an impact metric that depends on test conditions and velocity regime. The present theory links its thickness scaling to confinement-induced modification of the high-rate shear stiffness.

We test Eq. **9** against ballistic data for multilayer graphene (11–14), graphene oxide films under 1000m/s impact (15), and polymer thin films at comparable velocities (16) (Fig. 1). In all cases the model captures both the strong enhancement of penetration resistance in the ultrathin regime and the crossover toward a bulk plateau, supporting a general finite-size elastic mechanism that is observed across the systems considered here.

**Fig. 1.**
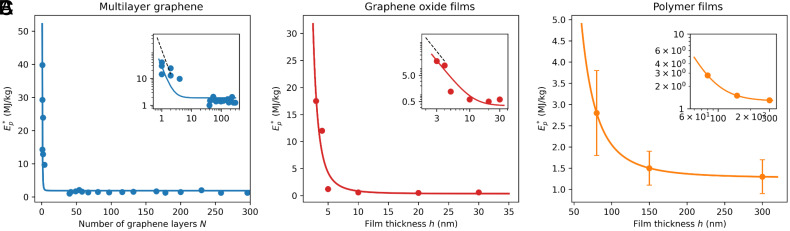
General inverse–cube thickness scaling of the specific penetration energy. (*A*) Multilayer graphene: specific penetration energy $E_{p}^{*}$ as a function of the number of graphene layers *N*, digitized from Fig. 4b of Bizao et al. (11). (*B*) Graphene oxide films under 1,000m/s impact (15). (*C*) Polymer thin films under 800m/s impact from ref. 16. Solid lines are fits to Eq. **9**. Dashed lines in the log–log *Insets* indicate the pure power-law trend $h^{-3}$.

The coefficient *B* measures the strength of confinement–induced stiffening. Its magnitude determines the thickness at which the finite–size contribution $Bh^{-3}$ overtakes the bulk term $E_{p,\infty }^{*}$. For graphene oxide films, the fitted value implies that already at h∼5nm the finite–size contribution exceeds the bulk penetration energy by more than an order of magnitude. *B* is a material-dependent coefficient proportional to $\beta^{\prime }/\rho$ that encodes nonaffine relaxation; more disordered materials are expected to exhibit larger *B*.

This behavior is predicted by microscopic elasticity: Long-wavelength nonaffine modes soften the shear modulus in disordered solids (3), hence their suppression due to confinement increases the rigidity. Experiments on ultrathin polymer films likewise report strong thickness-dependent elastic moduli (17, 18), consistent with confinement restricting accessible deformation modes. Suppressing these long-wavelength modes produces the $h^{-3}$ stiffening highlighted here.

Material-specific mechanisms (e.g., chain mobility, crystallinity, interlayer interactions) have also been proposed (16, 19). These may modify the prefactor or introduce corrections, while the $h^{-3}$ term reflects a generic elastic baseline.

The scaling $E_{p}^{*}\left(h\right)=E_{p,\infty }^{*}+Bh^{-3}$ is expected to hold most clearly in the elastic–inertial high-rate regime. At sufficiently high impact velocities, the loading time becomes shorter than structural relaxation times associated with molecular rearrangements, interlayer sliding, or chain mobility. The response therefore becomes effectively solid-like, and penetration resistance is controlled primarily by shear stiffness and acoustic impedance, consistent with $E_{p}^{*}\left(h\right)\propto G^{\prime }\left(h\right)$. In this regime projectile arrest occurs through localized plugging and rapid momentum transfer mediated by transient shear stresses rather than global bending. At lower velocities, viscoelastic or plastic relaxation mechanisms may mask the purely geometric confinement effect and produce deviations from simple power-law scaling. Inelastic processes may renormalize the prefactor or lead to deviations when they dominate; the observed agreement suggests the elastic term is leading-order in the ultrathin high-rate regime.

In conclusion, the inverse–cube thickness dependence of projectile penetration energy (followed by the bulk-like plateau) is shown to be a general consequence of finite-size elastic stiffening due to confinement-induced suppression of long-wavelength nonaffine modes. By linking ballistic impact resistance to microscopic nonaffine elasticity, the present results provide a common framework for disparate experimental observations across crystalline, amorphous, and polymeric thin films while leaving room for material-specific dissipative and failure mechanisms.

## Data Availability

There are no data underlying this work. Previously published data were used for this work (Data used in this work were extracted from previously published studies of ballistic impact on thin films, including multilayer graphene simulations and experiments (11–14), graphene oxide film impact experiments (15), and polymer thin-film impact measurements (16). These datasets were digitized from the published figures and reanalyzed to test the scaling relation proposed here).
